# Changes in LXR signaling influence early-pregnancy lipogenesis and protect against dysregulated fetoplacental lipid homeostasis

**DOI:** 10.1152/ajpendo.00449.2016

**Published:** 2017-04-18

**Authors:** Vanya Nikolova, Georgia Papacleovoulou, Elena Bellafante, Luiza Borges Manna, Eugene Jansen, Silvère Baron, Shadi Abu-Hayyeh, Malcolm Parker, Catherine Williamson

**Affiliations:** ^1^Women’s Health Academic Centre, King’s College London, London, United Kingdom;; ^2^Centre for Health Protection, National Institute for Public Health and the Environment, Bilthoven, The Netherlands;; ^3^Laboratoire Génétique Reproduction et Développement, Université Clermont Auvergne, Clermont-Ferrand, France; and; ^4^Institute of Reproductive and Developmental Biology, Imperial College London, London, United Kingdom

**Keywords:** nuclear receptors/liver X receptor, pregnancy, liver, lipid and lipoprotein metabolism, triglycerides

## Abstract

Human pregnancy is associated with enhanced de novo lipogenesis in the early stages followed by hyperlipidemia during advanced gestation. Liver X receptors (LXRs) are oxysterol-activated nuclear receptors that stimulate de novo lipogenesis and also promote the efflux of cholesterol from extrahepatic tissues followed by its transport back to the liver for biliary excretion. Although LXR is recognized as a master regulator of triglyceride and cholesterol homeostasis, it is unknown whether it facilitates the gestational adaptations in lipid metabolism. To address this question, biochemical profiling, protein quantification, and gene expression studies were used, and gestational metabolic changes in T0901317-treated wild-type mice and *Lxrab*^−/−^ mutants were investigated. Here, we show that altered LXR signaling contributes to the enhanced lipogenesis in early pregnancy by increasing the expression of hepatic *Fas* and stearoyl-CoA desaturase 1 (*Scd1*). Both the pharmacological activation of LXR with T0901317 and the genetic ablation of its two isoforms disrupted the increase in hepatic fatty acid biosynthesis and the development of hypertriglyceridemia during early gestation. We also demonstrate that absence of LXR enhances maternal white adipose tissue lipolysis, causing abnormal accumulation of triglycerides, cholesterol, and free fatty acids in the fetal liver. Together, these data identify LXR as an important factor in early-pregnancy lipogenesis that is also necessary to protect against abnormalities in fetoplacental lipid homeostasis.

pregnancy is a dynamic state involving profound changes in the hormonal milieu of the mother that then signal adaptations in maternal nutrient metabolism. These adaptations are necessary to ensure a continuous supply of essential metabolites to support the growth and the development of the fetus as well as to provide the mother with sufficient energy stores to meet the demands of pregnancy and prepare for lactation (15, 26). Pregnancy-induced changes in energy and nutrient metabolism follow a biphasic pattern. Early pregnancy is mainly anabolic since it is associated with augmented lipid deposition in maternal tissues as a consequence of maternal hyperphagia, increased de novo lipogenesis, and enhanced insulin sensitivity (15). In contrast, advanced pregnancy is an overall catabolic state involving augmented hydrolysis of stored lipids, hyperlipidemia, and insulin resistance (15).

Liver X receptors LXRα (*NR1H3*) and LXRβ (*NR1H2*) are oxysterol-activated nuclear receptors with key roles in the regulation of lipid metabolism. The two LXR isoforms share considerable sequence homology, but their tissue distribution differs: LXRα is highly expressed in liver, intestine, adipose tissue, and macrophages whereas LXRβ is ubiquitously expressed (31). LXR stimulates de novo lipogenesis by increasing the expression of sterol-regulatory element-binding protein-1c (SREBP1c), a master transcriptional regulator of fatty acid and triglyceride synthesis (32, 34). LXR also binds directly to the promoters of the lipogenic enzymes fatty acid synthase (FAS), stearoyl-CoA desaturase 1 (SCD1), and acetyl-CoA carboxylase 1 (ACC1; 9, 18, 38). Moreover, LXR plays a pivotal role in the regulation of cholesterol homeostasis: upon activation, it drives the efflux of cholesterol from nonhepatic tissues (e.g., macrophages and enterocytes), stimulates biliary excretion of the sterol in the liver, and reduces the intestinal absorption of luminal sterols (27, 30, 32, 40). However, there is limited understanding of how this nuclear receptor controls lipid metabolism in the physiological settings of pregnancy. Detailed knowledge of the molecular mechanisms responsible for lipid homeostasis during pregnancy is important as evidenced by the numerous reports demonstrating that failure to maintain the levels of circulating lipids and lipoproteins within physiological ranges throughout gestation results in maternal dyslipidemia and fetal metabolic complications such as macrosomia and increased risk of type 2 diabetes mellitus in adulthood (19, 23, 39).

We hypothesized that alterations in LXR activity could contribute to the gestational adaptations in lipid metabolism during pregnancy. Wild-type mice challenged with T0901317 (a potent and highly specific synthetic LXR agonist) throughout gestation and *Lxrab*^−/−^ mutants were used to examine the role of LXR in pregnancy metabolic adaptations. In the present study, we identify changes in LXR signaling as an important factor that influences early-pregnancy lipogenesis in mice. Our data also suggest that LXR protects against abnormalities in fetoplacental lipid homeostasis during murine pregnancy.

## MATERIALS AND METHODS

### 

#### Animal experiments.

C57BL/6 mice were purchased from Harlan Laboratories. LXR wild-type (WT) and *Lxrab*^−/−^ [LXR double knockout (DKO); model previously described (27)] mice were maintained on a mixed-strain background (C57BL/6:Sv129). All the mice were housed in a temperature- and light-controlled environment with 12:12-h light-dark cycles. Age-matched mice had free access to water and were fed a standard chow diet or chow supplemented with 0.012% T0901317 (T0901317 diet; Cayman Chemical; 34). Pregnant females were euthanized on *days 7, 10, 14*, and *18* of gestation. Tissues and sera were collected from chow-fed virgin controls and nonpregnant mice fed with a T0901317 diet for a period of 7, 10, 14, or 18 days. Mice, fasted for 4 h with free access to water, were euthanized by CO_2_ asphyxiation. All studies were conducted in conformity with the Public Health Service Policy on Humane Care and Use of Laboratory Animals, were approved by local animal ethics committees in King’s College London, and were authorized by the Home Office.

#### Lipid analysis.

Lipid extraction was performed as previously described (25). Briefly, frozen tissue fragments were homogenized in Hank’s balanced salt solution using TissueLyser II system (Qiagen, Manchester, UK) for 2 min at 30 Hz, and then the samples were centrifuged at 12,000 rpm for 15 min at 4°C. The supernatant was discarded, and the pellet was then resuspended in 500 μl of potassium phosphate lysis buffer with Triton X-100. The samples were then sonicated for 8 min at 4°C (a 30-s sonication phase followed by a 30-s resting phase) using a Bioruptor Plus system (Diagenode). Following a centrifugation at 10,000 rpm for 20 min at 4°C, the supernatants were transferred to clean glass containers and were stored at −80°C. Serum and tissue extracts were processed and tested in the laboratory of Dr. Eugene Jansen (Laboratory for Health Protection Research, National Institute of Public Health and the Environment, Bilthoven, The Netherlands). A Beckman Synchron LX20 chemistry analyzer (Beckman Coulter) and commercially available kits were used for the measurement of total cholesterol (467825; Beckman Coulter), HDL cholesterol (467820; Beckman Coulter), triglycerides (445850; Beckman Coulter), and free fatty acids [NEFA-HR(2) assay kit, Wako Diagnostics]; measurements were normalized to tissue protein content as previously described (25).

#### RNA isolation, cDNA synthesis, and quantitative real-time PCR analysis.

Total RNA was extracted using an RNeasy Mini Kit (Qiagen; liver and placenta samples) or RNeasy Lipid Tissue Mini Kit (Qiagen; adipose tissue) and then reverse transcribed into cDNA with random hexamers using SuperScript II Reverse Transcriptase (Thermo Fisher Scientific) in accordance with the manufacturers’ protocols. Real-time quantitative PCR reaction was performed on a Viia7 system (Thermo Fisher Scientific), in a 384-well assay format using SYBR Green Mastermix (Sigma-Aldrich). Relative mRNA levels were calculated using the comparative cycle threshold method normalized to cyclophilin. Primer sequences are listed in Supplemental Table S1 (all supplementary material for this article is accessible on the journal website).

#### Western blotting.

Total cell lysates were prepared using radioimmunoprecipitation assay (RIPA) buffer (Sigma-Aldrich) supplemented with a protease inhibitor cocktail (Sigma-Aldrich) as well as Phosphatase Inhibitor Cocktails 2 and 3 (Sigma-Aldrich). Samples were separated on a 12% SDS-polyacrylamide gel and transferred to a nitrocellulose membrane. Membranes were hybridized with rabbit anti-adipose triglyceride lipase (ATGL; 2138, 1:1,000; Cell Signaling Technology), rabbit anti-phospho-hormone-sensitive lipase (HSL; Ser563; 4139, 1:1,000; Cell Signaling Technology), rabbit anti-HSL (4107, 1:1,000; Cell Signaling Technology), mouse anti-LXRα (ab41902, 1:1,000; Abcam), rabbit anti-LXRβ (ab28479, 1:500; Abcam), and mouse anti-GAPDH (MAB374, 1:80,000; Millipore) antibodies at 4°C overnight to detect expression, followed by 1-h incubation at room temperature with goat anti-mouse (sc-2005, 1:4,000; Santa Cruz Biotechnology) and goat anti-rabbit (P0448, 1:2,000; Dako) peroxidase-conjugated antibodies. Proteins were detected by chemiluminescence (Millipore).

#### Statistical analysis.

All values are expressed as means ± SE for biological replicates. Unpaired two-tailed *t*-test was used for single comparisons whereas multiple comparisons were analyzed using multiple measures of ANOVA with Newman-Keuls post hoc testing.

## RESULTS

### 

#### Alterations in serum and hepatic lipids during mouse pregnancy.

To examine the impact of pregnancy on murine lipid metabolism, serum lipids were profiled in mice at different stages of pregnancy: *day 7* postcoitum, corresponding to early pregnancy (EP), and *days 10, 14*, and *18* postcoitum, taken to represent advanced pregnancy (AP). Virgin female mice were used as nonpregnant controls. During pregnancy, serum triglyceride concentrations were unchanged in early pregnancy and then gradually increased starting from *day 10* of mouse pregnancy onward (Table 1). In contrast, total cholesterol levels decreased in early pregnancy (at *day 7*) and were further reduced in advanced gestation. The same pattern was observed in the abundance of cholesterol in HDL. Serum levels of free fatty acids were not significantly altered during pregnancy. Since the liver functions as a metabolic hub that regulates all domains of lipid homeostasis (2, 34), the temporal changes in hepatic lipid profiles in pregnant mice were examined. In early pregnancy, hepatic triglyceride and cholesterol content was significantly increased whereas the levels of free fatty acids remained unchanged (Table 1). During advanced pregnancy, concentrations of hepatic triglyceride and cholesterol returned to preconception levels.

**Table 1.** Adaptations in serum and hepatic lipid profiles during mouse pregnancy

			Advanced Pregnancy		
	Nonpregnant, D0	Early Pregnancy, D7	D10	D14	D18
Serum					
Triglycerides, mmol/l	0.80 ± 0.04	0.76 ± 0.11	1.16 ± 0.06*	1.34 ± 0.16†	1.46 ± 0.13‡
Free fatty acids, mmol/l	0.81 ± 0.06	0.94 ± 0.09	1.07 ± 0.08	0.82 ± 0.09	0.93 ± 0.03
Total cholesterol, mmol/l	2.13 ± 0.12	1.80 ± 0.06‡	0.71 ± 0.05‡	1.33 ± 0.04‡	0.97 ± 0.04‡
HDL cholesterol, mmol/l	2.13 ± 0.06	1.70 ± 0.07‡	0.57 ± 0.05‡	1.32 ± 0.04‡	1.05 ± 0.04‡
Liver					
Triglycerides, µmol/g	990.11 ± 37.73	1,324.50 ± 119.98*	1,280.56 ± 89.90	1,058.96 ± 73.03	994.93 ± 143.46
Free fatty acids, µmol/g	222.63 ± 39.49	202.18 ± 26.80	286.68 ± 29.12	318.29 ± 31.14	203.59 ± 31.52
Cholesterol, µmol/g	54.93 ± 2.65	109.58 ± 14.45†	103.03 ± 13.24†	77.74 ± 5.20	83.31 ± 10.22

Values are means ± SE (*n* = 6–8). Total triglycerides, free fatty acids, cholesterol, and HDL cholesterol were measured in mouse serum, and total triglycerides, free fatty acids, and cholesterol were measured in hepatic lipid extracts.

* *P* < 0.05,

† *P* < 0.01,

‡ *P* < 0.001, comparison of pregnant groups [*days* (D) *7–18*] vs. nonpregnant group (D0). *P* values were determined by one-way ANOVA with Newman-Keuls post hoc testing.

#### Alterations in the hepatic LXR transcriptome during pregnancy.

**Fig. 1.**
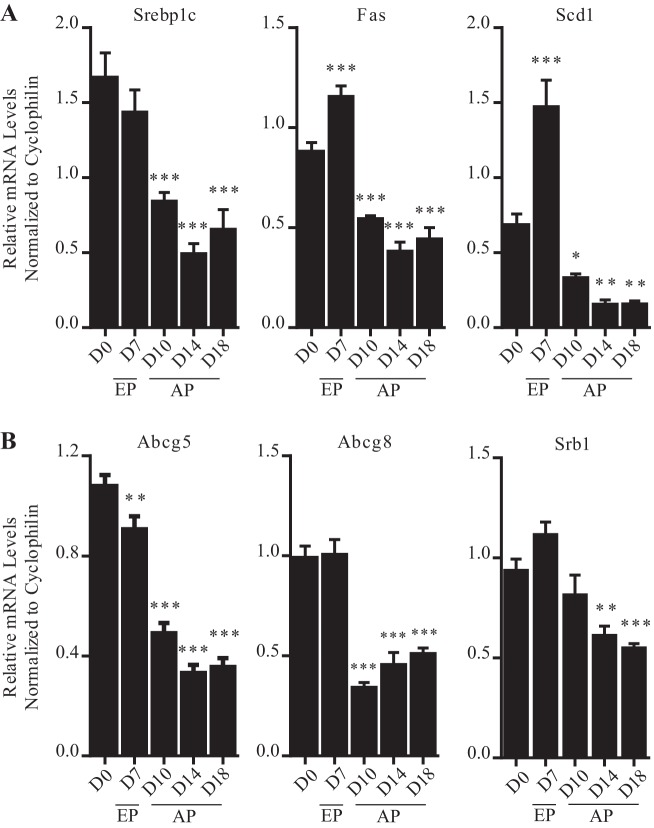
Increased expression of lipogenic liver X receptor (LXR) targets in mouse liver during early pregnancy. *A*: lipogenic genes. *B*: cholesterol homeostatic genes. Results are represented as means ± SE (*n* = 6–8); *P < 0.05, **P < 0.01, ***P < 0.001, comparison of pregnant groups [*days* (D) *7–18*] vs. nonpregnant group (D0). *P* values were determined by one-way ANOVA with Newman-Keuls post hoc testing.

Throughout pregnancy the expression of LXR target genes involved in hepatic lipogenesis follow a distinct biphasic pattern. In early pregnancy, consistent with the early-gestational increase in hepatic triglycerides, the transcript levels of the lipogenic targets *Fas* and *Scd1* were substantially raised (Fig. 1*A*). In contrast, during advanced pregnancy the mRNA abundance of *Srebp1c, Fas*, and *Scd1* was markedly diminished.

LXR controls hepatic cholesterol homeostasis by regulating the scavenger receptor B1 (SRB1), a membrane-bound glycoprotein that mediates the selective and efficient uptake of cholesteryl esters specifically from HDL, as well as the ATP-binding cassette G5 and G8 (ABCG5 and ABCG8) canalicular half-transporters, which form an obligate functional heterodimer that mediates the efflux of hepatic free cholesterol into bile (3, 21). Similar to the lipogenic genes, the cholesterol efflux genes *Abcg5, Abcg8*, and *Srb1* were also significantly downregulated during advanced pregnancy in mouse livers compared with nonpregnant controls (Fig. 1*B*). In contrast, the mRNA expression of the ATP-binding cassette A1 [*Abca1*; an LXR target gene encoding a transmembrane protein, which mediates the transfer of cholesterol and phospholipids to lipid-poor plasma carriers such as apolipoprotein A1 (4)] was significantly raised in early pregnancy and then returned to preconception levels during advanced gestation (data not shown).

In the liver, LXRα is the dominant nuclear receptor isoform (1). Immunoblot analysis revealed that the dynamic alteration in the expression of bona fide targets in the liver during pregnancy was not accompanied by any changes in the protein levels of LXRα (data not shown). The protein abundance of LXRβ also remained unaltered thus excluding a potential compensatory role.

Gene expression analysis revealed that pregnancy has no significant impact on the expression of either *Lxr* isoform or its targets in the duodenum [*Abca1, Abcg5, Abcg8*, and Niemann-pick c1 l1 (*Npc1l1*)] and primary blood monocytes [*Abcg1, Srb1*, ADP-ribosylation factor like 7 (*Arl7*), and LDL receptor (*Ldlr*)] (data not shown) thereby suggesting that changes in LXR activity are unlikely to affect intestinal cholesterol absorption and the reverse transport of sterols from extrahepatic tissues during pregnancy.

#### LXR remains transcriptionally active during pregnancy.

After confirming that changes in the protein availability of LXR could not explain the diminished mRNA levels of hepatic LXR targets during advanced-to-late pregnancy, we tested whether the reduced transcription of these genes could result from loss of LXR function during pregnancy. To pharmacologically activate LXR during gestation, pregnant mice were fed standard chow diet or T0901317-supplemented diet for 7, 10, 14, and 18 days following the identification of a copulatory plug. Nonpregnant female mice fed a T0901317 or chow diet for the same number of days were used as controls.

**Fig. 2.**
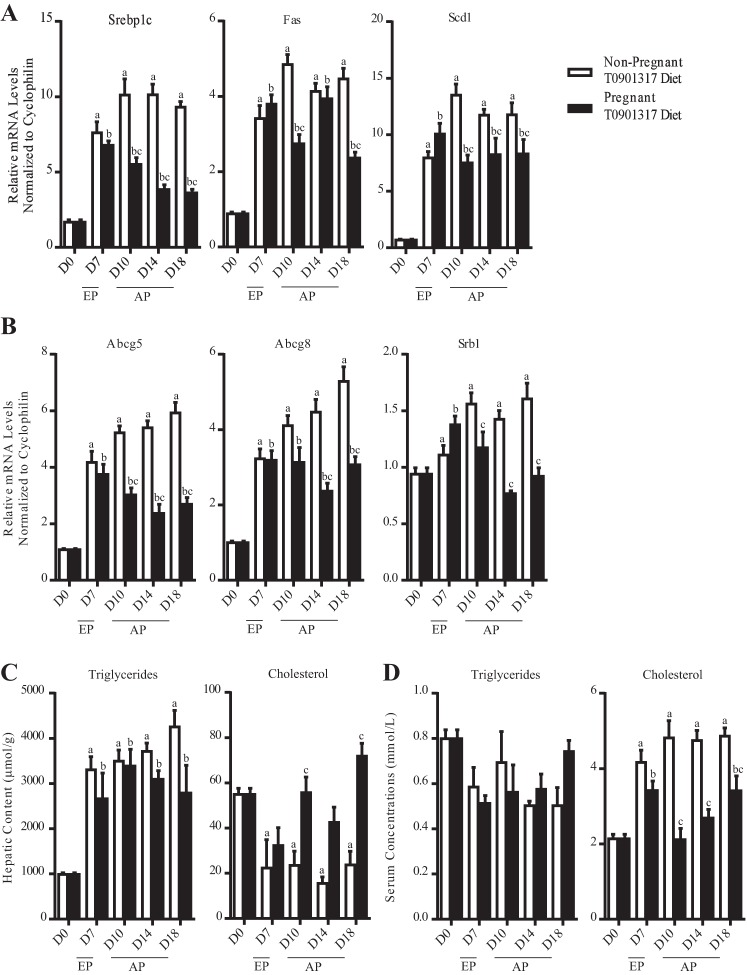
Pharmacological activation of LXR mimics early-pregnancy lipogenesis in mice. *A*: expression of lipogenic genes. *B*: expression of cholesterol homeostatic genes. *C*: total triglycerides and cholesterol measured in lipid extracts. *D*: total triglycerides and cholesterol measured in mouse serum. Results are represented as means ± SE (*n* = 6–8); ^a^*P* < 0.05, comparison of nonpregnant T0901317-fed groups [*days* (D) *7–18*] vs. nonpregnant group (D0); ^b^*P* < 0.05, comparison of pregnant T0901317-fed groups (D7-18) vs. pregnant group (D0); ^c^*P* < 0.05, comparison of pregnant T0901317-fed group vs. corresponding nonpregnant T0901317-fed group. *P* values were determined by one-way ANOVA with Newman-Keuls post hoc testing.

Comparison of the pregnant T0901317-fed groups vs. pregnancy-matched chow-fed groups revealed that the expression of LXR targets involved in lipogenesis (*Srebp1c, Fas*, and *Scd1*) and cholesterol efflux (*Abcg5* and *Abcg8*) was markedly increased. These data indicate that LXR is transcriptionally active during pregnancy and pharmacological activation of LXR is able to reverse the reduction in the mRNA levels of key metabolic genes in mouse liver in late pregnancy (Fig. 2, *A* and *B*). The gene expression changes were functionally confirmed by the significant increase in hepatic triglyceride levels and the reduction in hepatic cholesterol concentrations (Fig. 2*C*). Serum lipid profiling revealed that treatment of pregnant mice with T0901317 raises the levels of circulating cholesterol without affecting the levels of circulating triglycerides; these observations are in agreement with previous reports detailing the effect of this synthetic LXR agonist on nonpregnant mice (2, 34).

Activation of LXR contributes to early-pregnancy lipogenesis. To determine whether the adaptations in lipid metabolism observed during normal pregnancy are entirely LXR dependent and therefore completely reversed by the presence of T0901317, pregnant T0901317 mice were compared against nonpregnant diet-matched controls.

T0901317 mimics the gestational signal that promotes lipogenesis during early pregnancy. Gene expression analysis showed that the mRNA levels of the lipogenic LXR targets *Fas* and *Scd1* were not further upregulated in T0901317-fed mice in early pregnancy compared with diet-matched nonpregnant controls (Fig. 2*A*). Also, there were no differences in the hepatic triglyceride content of pregnant and nonpregnant mice challenged with the agonist-supplemented diet for 7 days (Fig. 2*C*).

During advanced pregnancy, however, the T0901317 agonist was not able to reverse the gestational changes in triglyceride homeostatic pathways in the liver. Specifically, from *day 10* onward the expression of *Srebp1c, Fas*, and *Scd1* was significantly reduced in pregnant mice administered with T0901317 compared with diet-matched nonpregnant controls (Fig. 2*A*). Interestingly, serum lipid profiling revealed that the presence of T0901317 abrogated the gestational increase in circulating triglycerides during advanced pregnancy (Fig. 2*D*).

Pharmacological activation of LXR had a limited effect on the gestational adaptations in cholesterol metabolism. Lipid profiling showed that the gestational increase in hepatic cholesterol levels on *days 7* and *10* of pregnancy were abrogated by the presence of T0901317. In contrast, this synthetic agonist was unable to reverse the gestational changes in the expression of cholesterol homeostatic genes in the liver as evidenced by the fact that the transcript abundance of the cholesterol transporters *Abcg5, Abcg8*, and *Srb1* was lower in pregnant T0901317-fed mice compared with nonpregnant T0901317-fed controls during advanced pregnancy (Fig. 2*B*). Administration of T0901317 to pregnant mice also did not interfere with the pregnancy-associated drop in serum cholesterol as evidenced by the fact that pregnant mice challenged with the agonist-supplemented diet had significantly lower circulating total cholesterol levels from *day 10* onward compared with diet-matched nonpregnant controls (Fig. 2*D*).

Overall, these results suggest that pharmacological activation of LXR mimics the early-pregnancy increase in lipogenesis. During advanced pregnancy, however, T0901317 appeared unable to compete with the gestational cues that signal the alterations in triglyceride and cholesterol metabolism, and therefore the patterns of gestational adaptations in lipid homeostasis were preserved throughout this period.

#### LXR is required for lipogenesis during pregnancy.

To delineate the functional importance of LXR in the control of the gestational adaptations in fatty acid and cholesterol metabolism and also to investigate to what extent the T0901317-induced effects during pregnancy were signaled directly via LXR, wild-type (WT) and *Lxrab*^−/−^ (LXR DKO) mice were studied at different stages of pregnancy. All the mice in this study were maintained on a C57BL/6 × Sv129 mixed background.

**Fig. 3.**
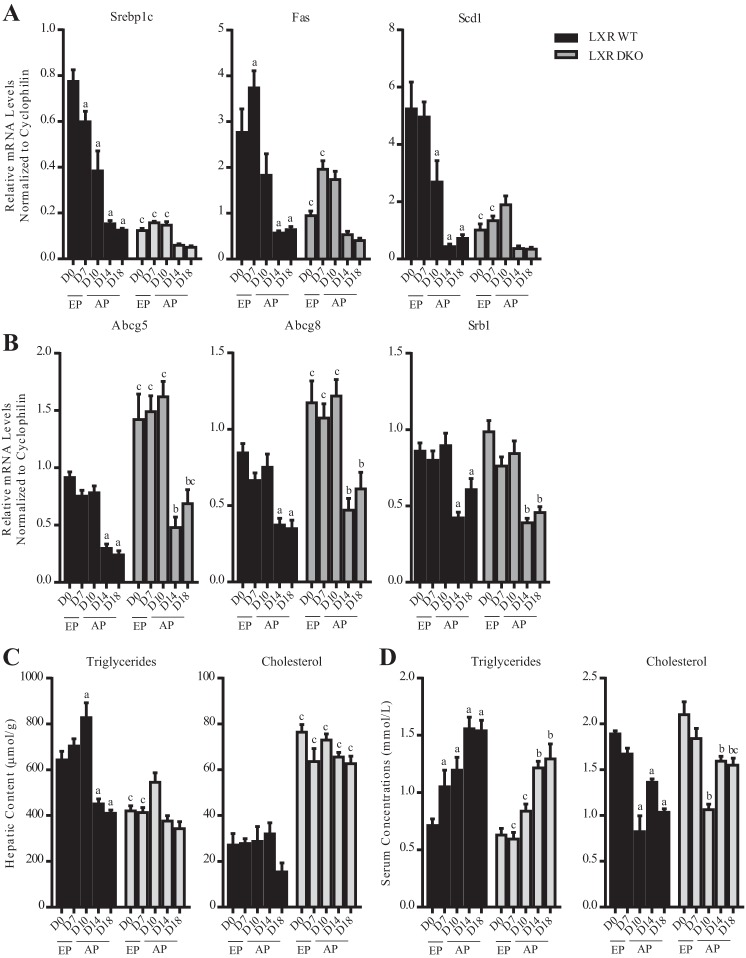
LXR is required for lipogenesis during early mouse pregnancy. *A*: expression of lipogenic genes. *B*: expression of cholesterol homeostatic genes. *C*: total cholesterol and triglycerides measured in hepatic lipid extracts. *D*: total triglycerides and cholesterol measured in mouse serum. ^a^*P* < 0.05, comparison of pregnant wild-type (WT) groups [*days* (D) *7–18*] vs. nonpregnant WT controls (D0); ^b^*P* < 0.05, comparison of pregnant LXR DKO groups (D7–18) vs. nonpregnant LXR DKO controls (D0); ^c^*P* < 0.05, comparison LXR DKO group vs. corresponding WT group. *P* values were determined by one-way ANOVA with Newman-Keuls post hoc testing.

In LXR DKO, the gestational adaptations in hepatic lipogenesis during both early and advanced gestation were lost as evidenced by the fact that no significant changes were detected in the expression of *Srebp1c, Fas*, and *Scd1* in the livers of pregnant mice compared with nonpregnant controls (Fig. 3*A*). Lipid quantification studies confirmed that in the absence of *Lxrab* all of the gestational changes in hepatic triglyceride levels were abolished (Fig. 3*C*). Serum biochemical profiling also showed that the early pregnancy-to-midpregnancy (*days 7* and *10*) increase in serum triglyceride levels was lost in mice lacking *Lxrab* (Fig. 3*D*).

#### LXR is not required for the gestational adaptations in cholesterol metabolism.

Study of WT and LXR DKO at different stages of pregnancy revealed that the absence of *Lxrab* had no impact on the gestational adaptations in cholesterol homeostasis. Both WT and LXR DKO mice showed significantly reduced expression of the cholesterol efflux transporters *Abcg5, Abcg8*, and *Srb1* on *days 14* and *18* of pregnancy (Fig. 3*B*). Even though LXR DKO mice had increased levels of hepatic cholesterol, there were no significant pregnancy-induced changes in cholesterol abundance either in the wild-type mice or in the mutants (Fig. 3*C*). Serum lipid profiling showed that in both WT and LXR DKO mice, the concentrations of total circulating cholesterol are significantly reduced during pregnancy from *day 10* onward (Fig. 3*D*).

#### Absence of LXR in the mother causes fetoplacental dyslipidemia.

**Fig. 4.**
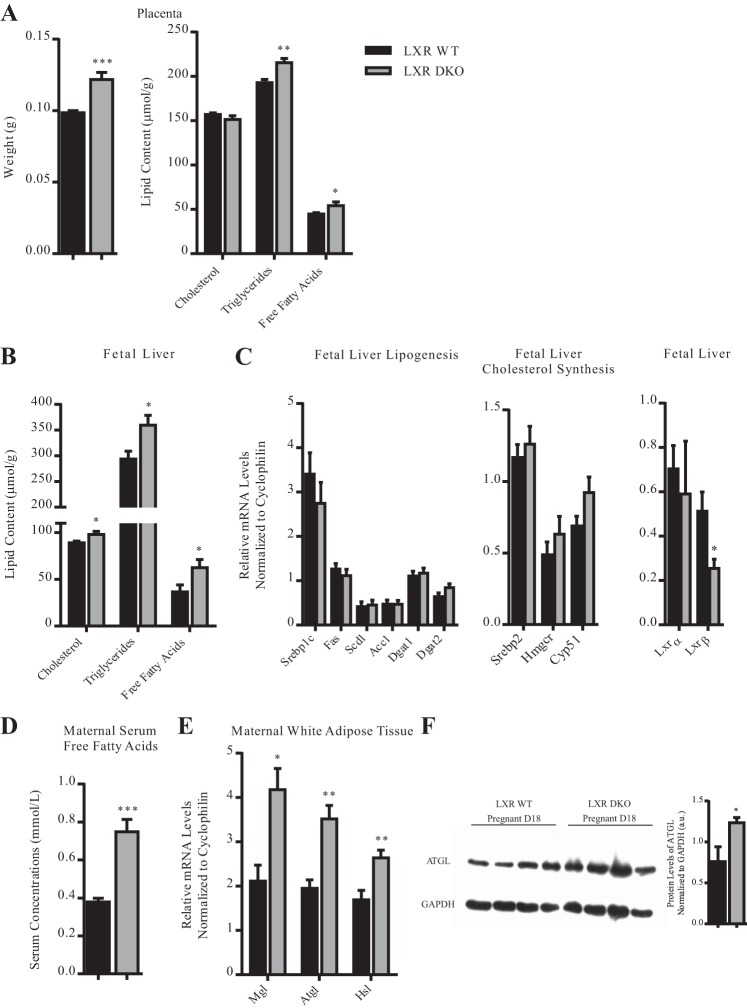
Gestational adaptations in LXR signaling protect against abnormalities in fetoplacental lipid metabolism in mice. *A*: placenta weight and total cholesterol, triglycerides, and free fatty acids measured in mouse placenta. *B*: total cholesterol, triglycerides, and free fatty acids measured in fetal hepatic extracts. *C*: mRNA expression of genes involved in de novo lipogenesis and cholesterol synthesis in fetal liver. *D*: levels of free fatty acid in maternal serum. *E*: expression of intracellular lipases in white adipose tissue. *F*: protein levels of ATGL in white adipose tissue; a.u., arbitrary units. Results are represented as means ± SE (*n* = 4–8). *P < 0.05, **P < 0.01, ***P < 0.001, comparison of wild-type (WT) vs. LXR DKO group. *P* values were determined by unpaired *t*-test.

It is recognized that exposure of fetuses to a dyslipidemic environment in utero causes enhanced fetal growth (19, 23, 39). Given that the absence of *Lxrab* interferes with maternal lipogenesis during pregnancy, we examined the effect of *Lxrab* deficiency on physiology and metabolism of the fetoplacental unit on *day 18* of gestation. Our results indicated that LXR DKO mice had increased placental weight accompanied with abnormal accumulation of triglycerides and free fatty acids in this organ (Fig. 4*A*). The *Lxrab*-heterozygous fetuses also had raised accumulation of cholesterol, triglycerides, and free fatty acids in their livers (Fig. 4*B*). Expression analysis revealed that fetuses heterozygous for *Lxrab* do not have significant changes in the mRNA levels of lipogenic genes [*Srebp1c, Fas, Scd1, Acc1*, and diglyceride acyltransferase 1 and 2 (*Dgat1 and Dgat2*)] and cholesterol biosynthetic genes [sterol-regulatory element-binding protein-2 (*Srebp2*), 3-hydroxy-3-methylglutaryl coenzyme A reductase (*Hmgcr*), and lanosterol 14α-demethylase (*Cyp51*)] in the liver (Fig. 4*C*). Interestingly, there were no differences in the transcript abundance of *Lxra*, the dominant isoform of LXR in the liver, in the heterozygous fetuses compared with the wild-type controls; the expression of *Lxrb*, however, was significantly downregulated.

Having determined that the fetoplacental hyperlipidemia detected on *day 18* of pregnancy in LXR DKO mice is unlikely to result from the augmented biosynthesis of lipids in the fetal liver, we tested whether this phenotype could arise from the deregulated lipid homeostasis in the mother. Biochemical analysis of maternal serum showed that in LXR DKO mice there was marked increase in the levels of free fatty acids on *day 18* of pregnancy (Fig. 4*D*).

Also, we examined whether the supraphysiological accumulation of free fatty acids in the serum of LXR DKO mice during advanced gestation could result from abnormalities in intestinal lipid absorption. mRNA quantification studies confirmed that *Lxrab* deficiency has no significant impact on the duodenal expression of key lipogenic targets [cluster of differentiation (*Cd36*), fatty acid transport protein (*Fatp4*), plasma membrane fatty acid binding protein (*Fabp-pm*), acetyl-CoA synthase 1 (*Acs1*), *Dgat1*, and *Dgat2*] in mice on *day 18* of pregnancy (data not shown). Similarly, there were no differences in the transcript abundance of genes involved in intestinal cholesterol absorption (*Abcg5*, *Abcg8*, and *Srb1*) between mutant mice and controls during advanced gestation; the expression of *Npc1l1* was significantly downregulated in the duodena of LXR DKO mice.

LXR plays a key role in the regulation of white adipose tissue metabolism (6); therefore we investigated whether the supraphysiological accumulation of fatty acids in the serum later in pregnancy could result from the deregulated lipolysis in the white adipose tissue of the mutant mice. Our results confirmed that expression of the intracellular lipases monoglycerol lipase (*Mgl*), adipose triglyceride lipase (*Atgl*), and hormone-sensitive lipase (*Hsl*) was considerably increased in the maternal white fat depots of LXR DKO mice (Fig. 4*E*). Protein quantification studies confirmed that the levels of ATGL in the adipose tissue of mice deficient in *Lxrab* were significantly increased (Fig. 4*F*); no changes in HSL phosphorylation were detected (data not shown).

## DISCUSSION

In the present study, we have identified that changes in LXR signaling influence early-pregnancy lipogenesis in mice. We have shown that pharmacological activation of LXR disrupts lipid biosynthetic pathways in the liver and prevents early-pregnancy hypertriglyceridemia. We have also demonstrated that absence of LXR deregulates maternal lipid homeostasis and enhances white adipose tissue lipolysis, which results in the increased accumulation of lipids in the fetal liver.

In this report we demonstrate that LXR targets contribute to early-pregnancy lipogenesis as evidenced by increased hepatic expression of *Fas* and *Scd1*, key enzymes involved in fatty acid biosynthesis, on *day 7* of mouse gestation and concurrent early-pregnancy accrual of hepatic triglycerides. Previous studies have demonstrated that SCD1 is the rate-limiting enzyme in the biosynthesis of monounsaturated fatty acids, which are major components of complex lipids such as diglycerides, triglycerides, and cholesteryl esters (22). Also, there is increased mRNA expression of *Scd1* in the fatty livers of mice with genetically induced obesity (11). Similarly, FAS catalyzes the biosynthesis of saturated fatty acids from simple precursors and, as such, has been categorized as the “gatekeeper” of de novo lipogenesis. Studies in rodents have demonstrated that FAS contributes to the storage of hepatic triglycerides and ~11% of the total triglyceride content in the liver is derived from de novo lipogenesis (12). FAS has an even more pronounced role in lipogenesis in the context of fatty livers as evidenced by the fact that *ob*/*ob* mice have enhanced *Fas* transcript abundance (17). On the basis of the presence of a molecular environment established as conducive to hepatic lipogenesis and also the direct quantification of the increment of lipogenic products (hepatic triglycerides) we have concluded that there is increased fatty acid biosynthesis in the livers of mice in early pregnancy. Moreover, the absence of raised free fatty acid and triglyceride concentrations in the serum of pregnant mice suggests that the hepatic lipids are unlikely to result from dietary lipids and peripheral fats stored in adipose tissue that flow to the liver by way of the plasma free fatty acid pool or from intestinally derived chylomicron remnants.

As pregnancy progressed, the transcript abundance of the tested lipogenic LXR targets was substantially reduced, and this change was reflected by the decrease in hepatic triglyceride concentrations. During advanced pregnancy, the levels of serum triglycerides increase significantly, and this change is not mirrored by an increase in the hepatic triglyceride content. Similarly, the molecular environment of the liver (i.e., the mRNA levels of *Srebp1*c, *Fas*, and *Scd1*) suggests reduced hepatic de novo lipogenesis in advanced murine pregnancy. These data are in agreement with previous studies (15). Moreover, it has been demonstrated that mice consume progressively greater amounts of food as gestation advances (20), and therefore it is likely that the elevated concentrations of circulating triglycerides result from the increased transfer of intestinally derived dietary lipids. These gestational adaptations occurred while the protein availability of both LXRα and LXRβ remained constant.

Having identified *Srebp1c*, *Fas*, and *Scd1* as factors involved in the dynamic regulation of triglyceride homeostasis during mouse pregnancy, we aimed to determine whether LXR is involved in control of these adaptations. Administration of the synthetic LXR agonist T0901317 confirmed that LXR is indeed transcriptionally active throughout gestation and therefore it could play a role in the gestational alterations in murine lipid homeostasis. Our data showed that the presence of T0901317 interfered with the early-pregnancy increase in *Fas* and *Scd1* transcription and subsequent triglyceride accumulation.

Our results indicate that the pregnancy signals that downregulate the expression of hepatic lipogenic factors during advanced pregnancy are potent enough to compete with T0901317-mediated activation of LXR. At present, there is limited understanding of how different maternal and placental hormones influence metabolic adaptations in the mother. Since the reduction in the expression of hepatic LXR targets parallels the completion of placenta formation, it is likely that factors of placental origin signal these adaptations directly, by binding to the LXR receptor itself, or indirectly, by initiating downstream signaling cascades that alter the phosphorylation state of LXR and thereby affect its activity.

Deletion of *Lxrab* abrogates all the changes in hepatic triglyceride homeostasis during murine pregnancy. This result indicates that during pregnancy, LXR is a key factor that relays the signals of reproductive hormones to the lipid metabolic network of the liver. Serum lipid profiling also demonstrated that mice lacking LXR have deregulated lipogenesis early in pregnancy, as evidenced by the fact that the increase in circulating triglycerides observed in wild-type mice on *gestational days 7* and *10* was lost in the global mutants. Gene expression analysis confirmed that in the absence of LXR, the transcript abundance of lipogenic genes in the liver does not exhibit any significant fluctuation in pregnancy. The results from our study could not exclude the possibility that gestational signals could reduce the expression of lipogenic targets independent of LXR in late pregnancy, as evidenced by the trends for diminished transcript abundance of *Srebp1c*, *Fas*, and *Scd1* in LXR DKO mice on *gestational days 14* and *18*.

LXR is a master regulator not only of fatty acid and triglyceride metabolism but also of cholesterol homeostasis (27, 30, 32, 40). Our data demonstrate that gestational adaptations in cholesterol metabolism are independent of LXR. Expression of the cholesterol efflux transporters *Abcg5, Abcg8*, and *Srb1* was downregulated during pregnancy independent of T0901317 challenge or *Lxrab* deletion. Furthermore, circulating total cholesterol concentrations were reduced during advanced pregnancy in all of our experimental models. Moreover, our data suggest that gestational changes in the expression of hepatic *Abca1* are unlikely to contribute to the pregnancy adaptations in cholesterol homeostasis. Also, the expression of this transporter in the duodenum remains unchanged throughout pregnancy (data not shown). These results are important as although the liver is the major source of plasma HDL, intestinal enterocytes also play a role in the biogenesis of this lipoprotein and 30% of the circulating HDL is derived from the intestine in an ABCA1-dependent manner (5). Thus we have concluded that changes in LXR signaling do not play a role in the gestational adaptations in cholesterol homeostasis. Further studies are necessary to elucidate the molecular mechanisms that influence cholesterol homeostasis during pregnancy.

Our data also revealed that defects in maternal lipid metabolism as a consequence of *Lxrab* deletion cause fetoplacental dyslipidemia in mice. Previous studies have demonstrated that LXR is expressed in white adipose tissue, where it is involved in the regulation of cholesterol, free fatty acid, and glucose metabolism (6). In vivo studies have reported that pharmacological activation of LXR increases adipose tissue lipolysis and fatty acid β-oxidation (37). We show that mice lacking both LXR isoforms have increased white adipose tissue lipolysis later in pregnancy, as evidenced by the raised maternal serum fatty acid levels and the upregulated expression of intracellular lipases in the white fat of these mice. Moreover, we demonstrated that *Lxrab* deficiency increases the protein levels of ATGL [a key lipolytic enzyme that controls energy homeostasis by catalyzing the rate-limiting step in triglyceride catabolism (13)] in the adipose tissue of pregnant mice. Our data suggest that changes in HSL phosphorylation are unlikely to contribute to the enhanced lipolytic response in adipose tissue of the *Lxrab*-mutant mice. Our conclusion that *Lxrab* deficiency promotes white fat lipolysis during advanced gestation is consistent with the observed in vivo phenotype (i.e., increased serum free fatty acid concentrations and reduced adipose tissue mass) as well as the obtained gene and protein expression data. Furthermore, the precise regulation of whole body lipid homeostasis depends on the continuous cross talk between liver and adipose tissue. Therefore we could speculate that the increase in white fat lipolysis in the pregnant LXR DKO mice could occur as a mechanism to compensate for the defects in hepatic lipogenesis in the mother and protect the fetus from any deficiencies in nutrient supply.

A key result from this study was that LXR DKO mice have increased placental weight and enhanced fatty acid and triglyceride accumulation in the placenta later in pregnancy. We also showed that fetuses from LXR DKO mice have increased free fatty acids, triglycerides, and cholesterol in the liver. Given that there was no induction of lipid biosynthetic pathways in the livers of the *Lxra*-heterozygous fetuses, it is likely that the fetal hepatic hyperlipidemia is a result of the deregulated maternal lipid homeostasis and impaired placental function. Consistent with this, a recent study has linked maternal hyperlipidemia with placental abnormalities and adverse fetal outcomes in cases of gestational diabetes mellitus (GDM; 35). Moreover, previous studies suggest that maternal adipose tissue lipolytic products are predominantly utilized as ketogenic substrates and the resulting ketone bodies get rapidly transferred to the fetal circulation, where they act as lipogenic precursors and energy fuels (15). Maternally derived free fatty acids are also able to cross the placenta and accumulate in the fetoplacental compartment (16). Our results excluded the possibility that the genetic background of the heterozygous fetuses could contribute to their hyperlipidemic phenotype since the mRNA expression of all fatty acid and cholesterol biosynthetic genes, as well as *Lxra*, was the same as in the wild-type controls. Previous studies have shown that gestational pathologies such as GDM and obesity, where maternal plasma levels of circulating lipids are supraphysiologically raised, are associated with fetal overgrowth (large-for-gestational age infants; 14, 28, 33, 36). Specifically, obese pregnant women typically have significantly increased levels of circulating triglycerides in combination with reduced HDL cholesterol levels and hyperglycemia as a consequence of exacerbated peripheral insulin resistance in the third trimester of gestation (29). The transplacental delivery of nutrients is quantitatively and qualitatively altered as a result of the increased total lipid content, and the accumulation of macrophages and proinflammatory mediators in the placentas of obese women leading to the birth of infants increased neonatal fat mass and body fat percentage (8, 24). Moreover, offspring of women with obesity and/or GDM not only are prone to adverse side effects at birth such as preterm delivery (10) and stillbirth (2) but also have a high risk of developing obesity, impaired glucose tolerance, and type 2 diabetes later in life (4, 7). All of these reports underscore the importance of thoroughly understanding the molecular determinants of alterations in maternal and fetal lipid metabolism during pregnancy to prevent immediate and long-term metabolic diseases. Our study identifies LXR as an attractive candidate that can be targeted with therapeutic interventions to reverse maternal and fetal dyslipidemia.

In conclusion, we present for the first time evidence that LXR is a factor essential for early-pregnancy lipogenesis in mice. We demonstrate that deletion of LXR not only interferes with the pregnancy adaptations in triglyceride metabolism in the liver but also causes fetoplacental dyslipidemia as a consequence of the raised maternal white fat lipolysis.

## GRANTS

This work was supported by the Wellcome Trust.

## DISCLOSURES

No conflicts of interest, financial or otherwise, are declared by the authors.

## AUTHOR CONTRIBUTIONS

V.N., S.B., S.A.-H., M.P., and C.W. conceived and designed research; V.N., G.P., E.B., L.B.M., and E.J. performed experiments; V.N. analyzed data; V.N. interpreted results of experiments; V.N. prepared figures; V.N. drafted manuscript; G.P., S.B., M.P., and C.W. edited and revised manuscript; C.W. approved final version of manuscript.
